# Stigma in Psychiatry: Impact of a Virtual and Traditional Psychiatry Clerkship on Medical Student Attitudes

**DOI:** 10.1007/s40596-021-01541-9

**Published:** 2021-10-14

**Authors:** Khalid Bazaid, Kevin Simas, Abdellah Bezzahou

**Affiliations:** 1 University of Ottawa, Ottawa, Ontario Canada; 2 Carleton University, Ottawa, Ontario Canada

**Keywords:** Psychiatry, Clerkship, COVID-19, Education, Attitudes

## Abstract

**Objective:**

The objective of the study was to assess the change in medical students’ attitudes towards psychiatry following a virtual clerkship experience compared to a traditional clerkship experience.

**Method:**

Ninety-seven medical students from the University of Ottawa were assessed pre- and post-clerkship on the ATP-30 (Attitudes Towards Psychiatry-30) measure. Cohorts of students were categorized as pre-COVID or during-COVID depending on when and how they experienced their clerkship (traditional or virtual). The total student response rate was approximately 48%. A quasi-experimental design was implemented, and non-parametric statistics were used to analyze the data.

**Results:**

Medical students’ overall attitudes towards psychiatry improved from pre- to post-clerkship, with the type of clerkship experience (traditional or virtual) having no significant impact on the magnitude to which attitudes improved.

**Conclusion:**

Implementation of a virtual clerkship in psychiatry did not deteriorate medical student attitudes towards psychiatry as a specialty, with both the traditional and virtual clerkship program enhancing students’ attitudes towards psychiatry favorably.

The coronavirus disease 2019 (COVID-19) pandemic has impacted many educational institutions worldwide by suspending most in-student activities. Many institutions had to abruptly move onto virtual teaching platforms. With little educational preparation and planning, it is unknown whether these virtual teachings have impacted a student’s learning experience. This is of particular importance within medical education given the in-person patient care component experienced during clerkships. Clerkships are important as they provide medical students with real-world and daily insight into the work, lifestyle, and responsibilities of physicians. Thus, understanding if a virtual curriculum has impacted a clerkship experience for medical students is crucial. No current study has been able to assess the impact of these virtual clerkships in comparison to traditional clerkships, with comparisons of pre-clerkship and post-clerkship changes in attitudes towards psychiatry. We were able to facilitate such analyses given our unique position having started data collection prior to COVID-19 and carrying on with data collection since.

A student’s attitude towards psychiatry as a medical specialty has been reliably reported to correlate positively with selecting psychiatry as a specialty [1], meaning clerkships may have a positive impact on their attitudes towards psychiatry. There are positive changes in attitudes towards psychiatry associated with a clerkship rotation in psychiatry [2, 3]. One study reported upon a meta-analysis of 26 studies; 16 studies (61%) demonstrated a post-clerkship improvement in attitudes towards psychiatry despite systematic differences in different medical school clerkships [4]. Furthermore, one of the only Canadian studies to examine the impact of a psychiatry clerkship on student attitudes towards psychiatry yielded positive attitude changes post-clerkship towards psychiatry, which were favorably enhanced by student clerkship rotation experience, especially if they were neutral towards psychiatry pre-clerkship [5]. Some studies report a positive correlation between a medical students’ positive clerkship experience and positive attitude changes towards psychiatry [6]. However, other studies report the opposite effect to also be true [7]. Another consistent finding reported across studies is the large percentage of medical students who share negative views about psychiatry as a career and as a subjectively exciting specialty, which typically does not change post-clerkship in psychiatry [2, 6, 8, 9]. This is to say, the true impact of psychiatry clerkships on students and their attitudes towards psychiatry is unclear. There is an inherent complexity about this literature, and a consensus is not apparent on the influential nature clerkships have on students and their attitudes towards psychiatry.

Since the initial emergence of COVID-19, clerkship rotations have been the most impacted part of a medical student’s education. For preclinical years, content learning migrated online, a goal that was in the works over the past few years and yielded alike or further improved learning outcomes [10]. However, for clerkship years, students were handed a roadblock that prevented active involvement in patient care during a pandemic due to the contagious nature of the virus, which would not be the case in an emergent natural disaster or mass casualty accident [11]. With this, the challenge that medical educational institutions faced was keeping patient experiences authentic for students in a virtual environment [11]. Of certainty is the unlikelihood of a return to pre-COVID teaching programs [10]. However, we see that it is possible to achieve certain teaching aims virtually, as evidenced through successfully implemented adaptive and self-directed student teaching [10]. Given this, investigating these new virtual clerkships and their impact remains important to ensure no compromise occurs in student clinical experience during their important formative educational years.

Thus, the objective of the present study was to assess the change in medical students’ attitudes towards psychiatry following a virtual clerkship experience compared to a traditional clerkship experience. We hypothesized that medical student attitudes towards psychiatry would deteriorate post-clerkship because of the transition to a virtual clerkship.

## Method

Approval to conduct this study and recruit medical students was obtained from the University of Ottawa research ethics board. From September 2019 until November 2020, third-year medical students from the University of Ottawa going through their psychiatry rotation were asked to participate in this study. Consent and study information were disseminated through the medical student society (Aesculapian Society), along with contact information to reach out to the Undergraduate Medical Education (UGME) psychiatry admin staff to express their interest. This was to ensure institutional policies regarding research with medical students were adhered to. Any students who did not respond or declined participation were not contacted. Physical or electronic copies of the consent form were disseminated to students who expressed interest. Consent to participate was provided verbally or electronically via email correspondence.

After initial dissemination of study information, communication with students was managed by the University of Ottawa UGME psychiatry admin staff to ensure no conflict of interest. Students were contacted by UGME admin staff at three time points: once for the initial recruitment process, a second time to collect consent and administer the questionnaire within the first week of clerkship, and a final time to readminister the questionnaire post-clerkship. Students were advised participation was voluntary, withdrawal from study participation could occur at any time, and that the decision to participate or to withdraw would have no bearing on their clerkship evaluation. Either physical or electronic copies of the questionnaire were completed. The survey questionnaire administered was the “Attitudes Towards Psychiatry-30” (ATP-30) questionnaire. The recruitment and data acquisition procedure remained the same pre-COVID and during-COVID, except for the administration of the consent information and questionnaire, which was provided and administered electronically during-COVID.

The ATP-30 is a self-administered questionnaire. It is a commonly used, standardized, and validated measure of medical students’ attitudes towards psychiatry as a medical discipline [12]. The measure is comprised of 30 items. Some items are positively coded being scored on a 5-point Likert scale ranging from 1 (*strongly disagree*) to 5 (*strongly agree*). Some items are negatively coded such that items range from 1 (*strongly agree*) to 5 (*strongly disagree*). Scoring of the ATP-30 involves the summation of all question responses and their associated value, with higher final ATP-30 scores indicating more positive attitudes towards psychiatry.

Our study began with a sample of *N* = 202 third-year medical students who were taking their mandatory psychiatry clerkship and consented to participate. Data was collected at the pre-clerkship and post-clerkship time points. Based on data eligibility criteria, 105 participants were excluded from analyses due to incomplete data at either time point. The final sample size was 97 students. There was also a low response rate (26.7%) for demographic data from the 202 students. The collected data spans from September 2019 until November 2020. Participants were separated into a pre-COVID cohort (*N* = 46) if they rotated between September 2019 and February 2020, and a during-COVID cohort (*N* = 51) if they rotated between July 2020 and November 2020. The pre-COVID cohort completed a traditional clerkship, and the during-COVID cohort completed a virtual clerkship.

The clerkship structure was remodeled and restructured due to limitations imposed by the pandemic. Psychiatry clerkships were held at different university-affiliated health care sites that offered psychiatric services. The traditional pre-COVID psychiatry clerkship program lasted for 6 weeks, beginning with a 1-week in-person bootcamp where students received courses on psychiatric topics prior to rotation. This was followed by 5 weeks of psychiatry rotations through different subspecialties: 3 weeks in adult psychiatry, 1 week in geriatric psychiatry, and 1 week in child and adolescent psychiatry. Students also had 4–5 on-call shifts where they were exposed to emergency psychiatry. The during-COVID psychiatry clerkship program eliminated some traditional clerkship components, and some of the rotations were also initially shortened because medical students were pulled from hospitals at the start of the pandemic. Throughout the virtual curriculum, rotations also lasted 6 weeks with reduced clinical time. The original 1-week in-person bootcamp was replaced by 2 weeks of online lectures, and the 5 weeks of in-person rotations through different subspecialties were replaced by 4 weeks of virtual rotations. Throughout the virtual clerkship, didactic lectures were delivered to students via Zoom calls. All clinical virtual rotations involved students attending clinical cases with their preceptors using safe virtual telemedicine portals (video or phone consultations). To supplement the reduced clinical time, students were expected to complete e-modules. Students were also limited in their exposure to various subspecialties due to the interruption of many psychiatric services.

We implemented a quasi-experimental design to examine differences in student attitudes towards psychiatry as measured per the ATP-30 between the pre-COVID and during-COVID cohorts across pre-clerkship and post-clerkship. We conducted a Mann–Whitney *U* test and Wilcoxon signed-rank test using SPSS (SPSS version 27) to assess total ATP-30 score differences from pre-clerkship to post-clerkship for both cohorts. As a result of the low response rate for demographic data, missing data analyses were not conducted. Statistical assumptions were met as our dependent variable (ATP-30 scores) was measured on an ordinal basis and our independent variable consisted of two categorically different groups of students assessed across pre- and post-clerkship within or between groups, while maintaining relative independency of each observed data point. Furthermore, for the Mann–Whitney *U* test, similar distribution shapes per independent group comparison were ensured. Lastly, for the Wilcoxon signed-rank test, the determination of symmetry of the distribution of differences scores for all applicable comparisons was tested and affirmed through visual boxplot examinations. Given these results, we were able to proceed forward with the analyses.

## Results

Of the *N* = 202 students, 97 completed all ATP-30 questions both pre-clerkship and post-clerkship (complete response rate of approximately 48%). Pre-clerkship ATP-30 scores ranged from 95 to 147, and post-clerkship ATP-30 scores ranged from 81 to 148. For the pre-COVID cohort, pre-clerkship ATP-30 scores ranged from 98 to 147 and post-clerkship ATP-30 scores ranged from 90 to 148. As for the post-COVID cohort, pre-clerkship ATP-30 scores ranged from 95 to 142 and post-clerkship ATP-30 scores ranged from 81 to 143. Of the 97 students with complete ATP-30 data on both pre-clerkship and post-clerkship, *N* = 46 (47.4%) were in pre-COVID cohort and *N* = 51 (52.6%) were in the during-COVID cohort.

Both the pre-COVID and during-COVID cohort were separately assessed on overall ATP-30 scores from pre-clerkship to post-clerkship (Table 1), with median differences calculated from pre-clerkship to post-clerkship. A Wilcoxon signed-rank test indicated that ATP-30 scores were higher post-clerkship (*Mdn* = 124) than pre-clerkship (*Mdn* = 117) for the pre-COVID cohort, *Z* = 3.025, *p* = 0.002, *d* = 0.45. Post-clerkship ATP-30 scores (*Mdn* = 119) were also higher than pre-clerkship ATP-30 scores (*Mdn* = 113) for the during-COVID cohort, *Z* = 3.578, *p* < 0.001, *d* = 0.50.

**Table 1:** Within-cohort and between-cohort ATP-30 score differences

Cohort	Median^a^		*P*-value
	Pre-clerkship	Post-clerkship	
Pre-COVID	117	124	.002*
During-COVID	113	119	< .001*
*P*-value	.034*	.052	

This table demonstrates change in ATP-30 (Attitudes Towards Psychiatry-30) scores for each respective cohort from pre- to post-clerkship with the Wilcoxon signed-rank test indication for significance. The table also demonstrates direct comparison between cohorts and their respective pre-clerkship ATP-30 score and post-clerkship ATP-30 score with the Mann–Whitney *U* test indication for significance

^*^Significance < 0.05

^a^Median = Median ATP-30 score

The magnitude of the change in ATP-30 scores between the two cohorts from pre-clerkship to post-clerkship was also assessed, utilizing a Mann–Whitney *U* test to compare the difference in scores (post-clerkship ATP-30 score minus pre-clerkship ATP-30 score). Median ATP-30 difference scores for the during-COVID cohort (*Mdn* = 5) did not significantly differ from mean ATP-30 difference scores for the pre-COVID cohort (*Mdn* = 5.5), *U* = 1245, *p* = 0.603, *d* = 0.05.

## Discussion

This is the only present study to date examining the impact of a virtual psychiatry clerkship on medical student attitudes towards psychiatry. This study is unique in that it is also able to directly compare this virtual psychiatry clerkship curriculum impact to the traditional curriculum impact on student attitudes towards psychiatry. Given psychiatry is a specialty where direct patient contact is crucial in diagnosis and treatment, the results of the study provide useful insight into future direction with clerkship curriculum programming. Although our hypothesis was not supported, we determined that overall student attitudes towards psychiatry improved post-clerkship, regardless of the traditional or virtual clerkship. Furthermore, there were no significant differences in the magnitude of improved student attitudes between the pre-COVID and during-COVID cohorts from pre- to post-clerkship, which suggests that the virtual and traditional clerkships were equally effective at improving student attitudes.

One limitation of the study is the extent to which these results are generalizable given institutional difference in clerkship programming. As of present, with most in-person rotations having resumed, the generalizability of the data may also be scrutinized. Furthermore, there is a risk of potential sampling bias given all data is from a single institution. The low complete response rate also introduces a risk of bias into the results, and given an even lower complete response rate for demographics, a missing data analysis comparison was not feasible to determine if any significant differences existed between students who had complete and non-complete data. Thus, this increases the risk for bias in our dataset further, and consequently could impact the validity of the results. Another limitation is that the study could not employ an experimental design with a true control group given the variability in cohort rotations throughout the year. Additional limitations include the risk of socially desirable responding, although anonymity in responses was used to prevent this, as well as a lack of self-check questions to ensure students were reading and answering the questions thoroughly and honestly.

The current study has shown that since the COVID-19 pandemic began and resulted in significant changes to the medical clerkship programming structure, those changes have not significantly deteriorated student attitudes towards psychiatry, rather seeming to have the same effect in improving students’ attitudes towards psychiatry post-clerkship experience.
